# Laparoscopic repair of intrathoracic kidney associated with giant congenital diaphragmatic hernia: an infant case report and literature review

**DOI:** 10.3389/fped.2024.1499644

**Published:** 2024-12-06

**Authors:** Ze Ji, Zhen Zhao, Hongwei Xi, Hongxia Ren

**Affiliations:** ^1^ Department of Neonatal Surgery, Shanxi Provincial Children’s Hospital, Taiyuan, China; ^2^ Department of Pediatrics, Shanxi Medical University, Taiyuan, China; ^3^ Department of General Surgery, Shanxi Provincial Children's Hospital, Taiyuan, China

**Keywords:** intrathoracic kidney (ITK), congenital diaphragmatic hernia (CDH), laparoscopy, infant, case report

## Abstract

**Background:**

Intrathoracic kidney (ITK) is a rare congenital disease, with only about 40 pediatric cases reported worldwide to date. ITK associated with congenital diaphragmatic hernia (CDH) is even rarer, and we report a case of an infant with ITK combined with a giant CDH.

**Case description and management:**

A six-month-old male infant was hospitalized due to “vomiting for 4 days”. The child's parents sought a definitive diagnosis and treatment to alleviate the child's suffering. Following a series of examinations and laboratory tests, we determined the child had ITK combined with CDH. We treated the condition laparoscopically, repairing the diaphragmatic defect and securing the kidney to the posterior wall of the abdomen. After a two-year follow-up period, the child exhibited no significant discomfort.

**Conclusions:**

Infantile ITK combined with giant CDH is relatively rare and the etiology is unclear. When symptoms of pneumonia, gastrointestinal obstruction or genitourinary tract occur, surgical intervention is necessary. Laparoscopic reduction of the ectopic kidney and repair of the giant diaphragmatic hernia is a minimally invasive and effective surgical approach.

## 1 Introduction

Intrathoracic kidney (ITK) is a rare congenital condition where the kidney is located above the diaphragm and enters the posterior mediastinum. The incidence is approximately 1/10,000, with a predilection for the left side and a male predominance (1). ITK is the rarest form of ectopic kidney, accounting for about 5% of cases (2). To date, there have been about 200 reported cases worldwide, including more than 40 pediatric cases (1, 3).

Upon a thorough review of the existing literature on ITK, we found that there is a significant dearth of reports focusing on the occurrence of ITK in conjunction with CDH in infants. This report aims to address this gap by presenting a case study of an infant diagnosed with left-sided ITK accompanied by a substantial CDH. We recount our successful application of laparoscopic surgery for the repair of the diaphragmatic hernia and the repositioning of the ectopic kidney, thereby contributing novel insights to the medical community.

The objective of this paper is not only to highlight the clinical manifestations and therapeutic approaches for the combined presentation of ITK and CDH but also to emphasize the value of minimally invasive surgical techniques in such rare and complex pediatric cases. We present this article in accordance with the CARE reporting checklist.

## 2 Case presentation

### 2.1 Basic information

The patient is a male infant, aged 6 months and 11 days, admitted to the hospital mainly due to “vomiting for 4 days”. The child began vomiting without any obvious cause 4 days prior to admission. The vomiting was non-projectile, and the vomitus was white, with no clear relationship to food intake. The child had poor appetite and intermittent fever, with the highest body temperature reaching 37.5°C. After the onset of the disease, the child received fluid replenishment and other symptomatic treatments at a local hospital, but the symptoms did not significantly improve; he was then transferred to our hospital for further diagnosis and treatment. The child has been in good health in the past, with no history of special diseases. The child is the firstborn and the second delivery, with a gestational age of 38 weeks, born by vaginal delivery, with a birth weight of 3,100 g. The child's mother had no special pregnancy or childbirth history during pregnancy. His family has no history of ITK or CDH.

### 2.2 Preoperative condition

Upon admission, the physical examination revealed that the child's mental state was fair, with no signs of respiratory distress or cyanosis around the mouth. Breath sounds in the left lower lung were weaker. The apical impulse of the heart was slightly shifted to the right, which was not normal. And the percussion of the left chest produced dull sounds. Abdominal examination did not reveal any significant bowel sounds, the abdomen was not distended, flat with soft abdominal muscles. And there was no tenderness or rebound pain, and no significant mass was palpable.

**Figure 1:**
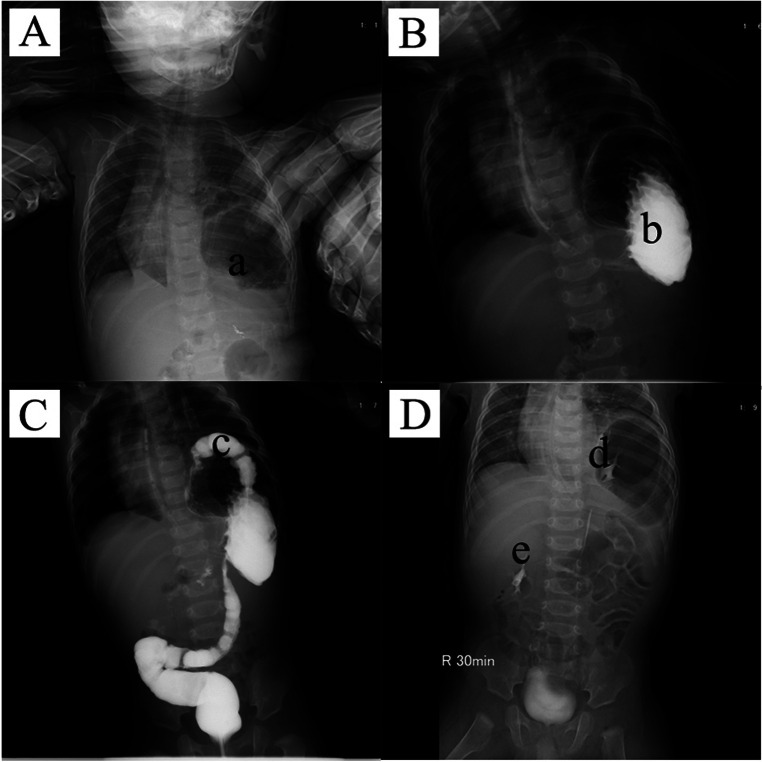
Preoperative examination of the child. **(A)** Chest X-ray. **(B)** Upper gastrointestinal series. **(C)** Lower gastrointestinal series. **(D)** Intravenous pyelography. a: huge gastric bubble shadow; b: folded stomach; c: transverse colon; d: the left kidney; **e**: the right kidney.

The auxiliary examination methods and results are as follows. Chest x-ray indicated: The left lung was compressed, there was a huge gastric bubble shadow in the left thoracic cavity, and the mediastinum was mildly shifted to the right (Figure 1A). Upper gastrointestinal series suggested: The stomach was folded, with the pylorus and gastric fundus entering the thoracic cavity, and there was obstruction at the gastric outlet (Figure 1B). Lower gastrointestinal series suggested: The transverse colon entered the left thoracic cavity (Figure 1C). Intravenous pyelography suggested (Figure 1D): The left kidney was located in the left thoracic cavity, with the renal axis slightly rotated and the renal pelvis forked. Bilateral ureters were intermittently displayed, with normal course, the bladder was well filled, and the excretory function of both kidneys was good.

**Figure 2:**
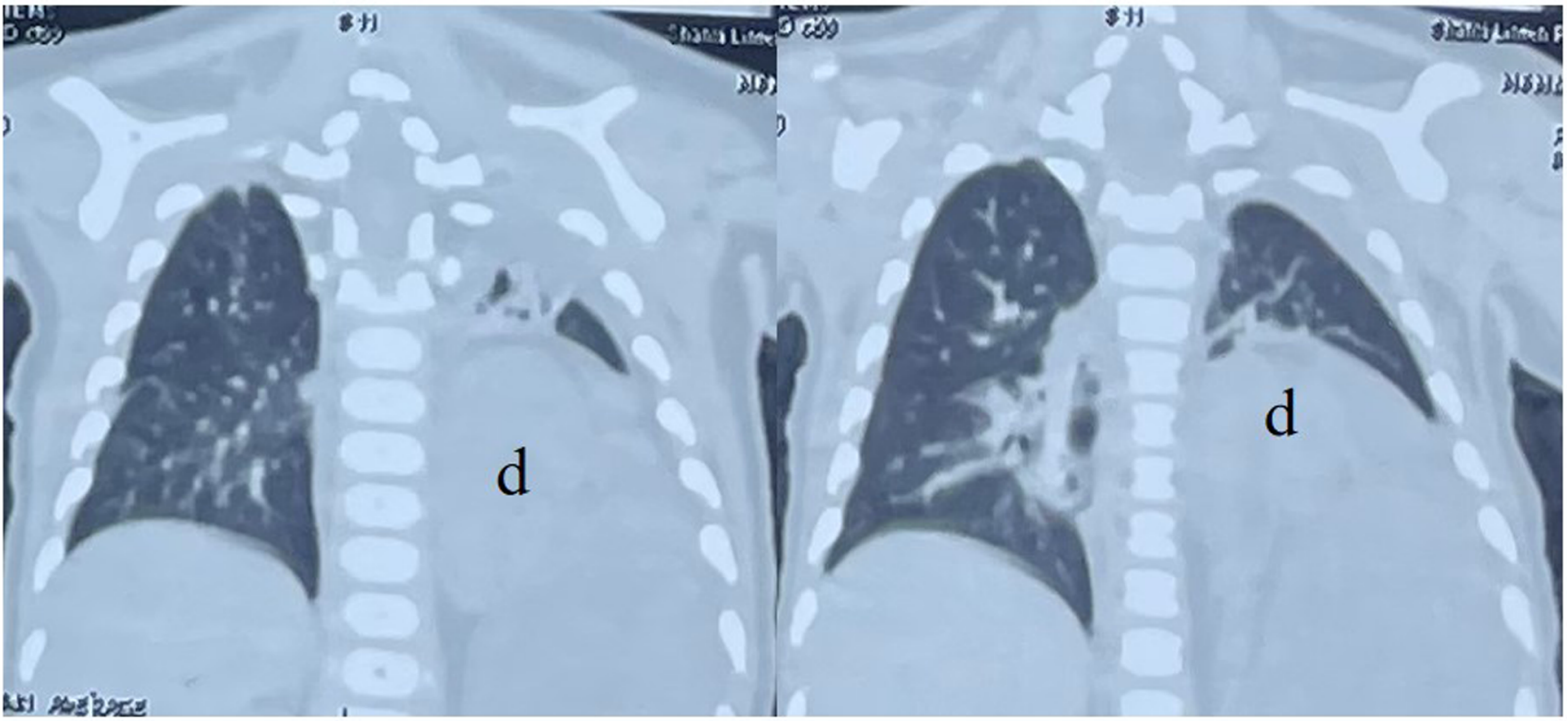
Chest CT scan. The left lung was compressed. d: the left kidney.

Chest CT scan indicated: The left kidney was located within the thoracic cavity, the mediastinum was displaced, and the left lung was compressed (Figure 2).

Urological Ultrasound: The left kidney, measuring 6.1 × 2.8 cm, is located superior to the spleen, approximately between the 7th and 10th intercostal spaces along the scapular line. It exhibits a normal morphology with a smooth capsule, clear differentiation between the cortex and medulla, and the collecting system is centralized without visualization of the ureters. Renal function tests reveal: Urea: 2.0 mmol/L, Creatinine: 51 µmol/L. These results suggest that the function of the left kidney remains good.

### 2.3 Diagnosis

(1) Left-sided ITK; (2) Left-sided giant CDH.

### 2.4 Surgical approach

Laparoscopic reduction of the ITK and repair of the giant CDH.

### 2.5 Intraoperative observations and surgical procedures

**Figure 3:**
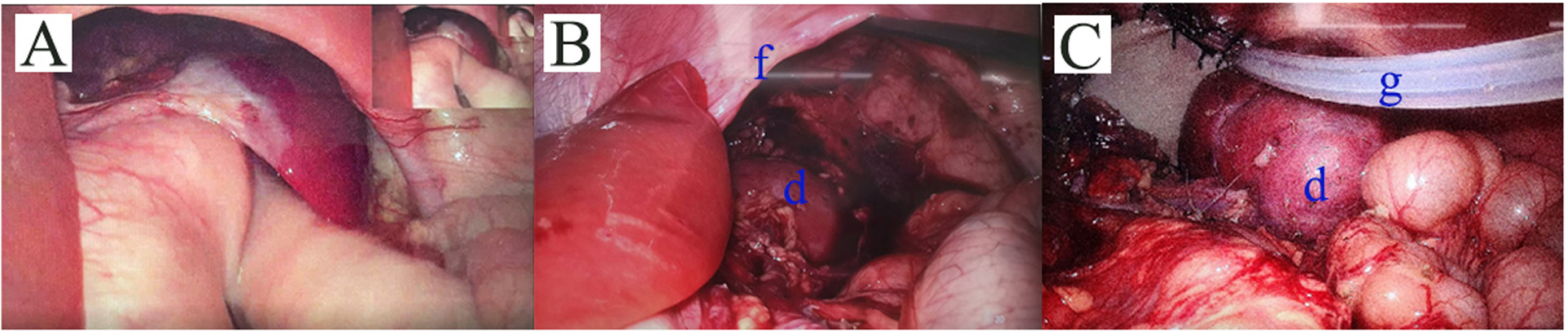
Intraoperative Observations. **(A)** The stomach and omentum were severely adhered to the diaphragmatic defect. And the giant hernia ring was visible. **(B)** The left thoracic kidney was visible, with the renal hilum showing no signs of rotation and was covered by the renal capsule. The anterior edge of the diaphragm was clearly visible, while the posterior edge was not seen. **(C)** Bovine pericardium patch was used to suture the diaphragmatic defect. The kidney was then fixed in a retroperitoneal position. And we placed a catheter. d: the left kidney; f: anterior edge of the diaphragm; g: the drainage tube.

The surgery was performed with the patient in the supine position. A 10-millimeter incision was made on the left side of the navel, and an appropriately sized trocar was inserted. After establishing pneumoperitoneum, the laparoscope was introduced. We then made a 5-mm incision in the left mid-abdomen, a 3-mm incision in the left upper abdomen, and a 3-mm incision in the right upper abdomen. Subsequently, we inserted appropriately sized trocars to place the operating forceps. Upon exploration, we observed that the stomach and omentum were severely adhered to the diaphragmatic defect. After lying the adhesions, the hernia ring was visible, with the spleen, stomach, small intestine, colon, and omentum herniated into the thoracic cavity (Figure 3A). We believed the patient had a condition of inadequate fixation of the mesentery. The diaphragmatic defect measured 8.0 cm by 5.0 cm. No distinct posterior margin of the diaphragm was observed at the site of the defect.

Posterior to the thoracic cavity, the left kidney was visible, with the renal hilum showing no signs of rotation and was covered by the renal capsule (Figure 3B).

After fully mobilizing the perirenal tissue, the renal hilum vessels, and the adrenal gland, the kidney was repositioned into the abdominal cavity. Bovine pericardium patch was used to suture the diaphragmatic defect. The kidney was then fixed in a retroperitoneal position (Figure 3C). The drainage was visible. We placed a drainage in the left anterior chest at the end of procedure, with the end located above the diaphragmatic defect. The drainage tube opening was situated in the existing incision in the left upper abdomen and was removed after 72 h. This surgery took a total of 340 min.

### 2.6 Postoperative condition

**Figure 4:**
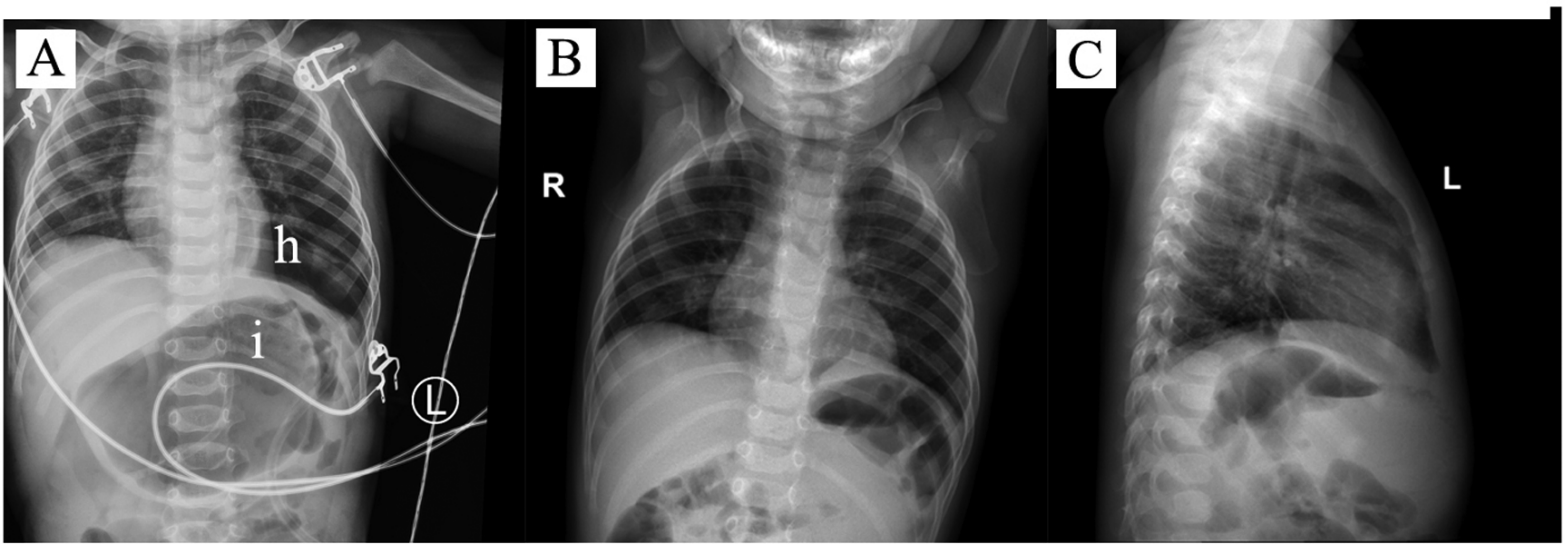
Postoperative Condition. **(A)** Postoperative Day 1 chest X-ray; **(B)** and **(C)** Postoperative Day 30 chest X-rays. h: an abnormally transparent shadow in the lower lobe of the left lung; i: large gastric bubble shadow.

On the first postoperative days, chest x-rays revealed: an abnormally transparent shadow in the lower field of the left lung and a large gastric bubble shadow (Figure 4A). It was considered that the abnormal transparency was due to a sealed cavity formed by the hernia sac, with residual gas that had not been completely expelled. The child exhibited no abnormal symptoms, and no treatment was given.

The child was discharged smoothly on the 8th postoperative day. One month later, during the outpatient follow-up, the chest x-ray showed the transparent shadow had disappeared (Figures 4B,C); the abdominal ultrasound revealed that the left kidney was located in the abdominal cavity with no abnormalities in size or morphology; the child exhibited no abnormal symptoms.

Postoperative telephone follow-ups at 6 months and at 1 year and 2 years, indicated that the child was living healthily with no abnormal symptoms. The patient has recovered and will continue to follow up.

## 3 Discussion

The pathogenesis of ITK is currently unclear. ITK may be due to accelerated ascent of the kidney or delayed closure of the diaphragm during the development process, or it may be caused by underdevelopment of the pleura and peritoneum (4). Some scholars believe that the interaction between the metanephros and the mesonephros, with the metanephros migrating cranially and the mesonephric tissue migrating in the opposite direction, and the delayed regression of the mesonephric tissue, leads to elongation of the nephric duct, resulting in an ectopic kidney in the thoracic cavity. Currently, it is widely believed that the thoracic kidney may be related to morphological abnormalities, abnormal rotation, elongation of the ureter, and a higher origin of the renal blood vessels (5).

ITK can be categorized into four types: ITK with diaphragmatic closure; ITK with diaphragmatic eventration; ITK with traumatic diaphragmatic rupture and ITK with CDH (6). The case we report in this instance falls under the fourth type, that is, an infant with ITK combined with a massive CDH, accompanied by symptoms of gastrointestinal obstruction.

CDH is a condition arising from the incomplete fusion of the pleuroperitoneal folds during the early stages of fetal development, leading to the herniation of abdominal organs into the thoracic cavity, with an incidence rate of 1/3,300, and a higher occurrence in males compared to females, with a ratio of about 10:7 (7). The posterolateral diaphragmatic hernia, known as Bochdalek hernia, is the most prevalent form of diaphragmatic hernia, mostly occurring on the left side (8). Symptoms of diaphragmatic hernia can vary widely depending on the size of the defect in the diaphragm. Individuals with a small defect may never experience symptoms and remain undiagnosed throughout life; whereas those with a larger defect can suffer from lung compression due to the herniation of abdominal organs into the thoracic cavity, which can impair breathing, potentially cause mediastinal shift, and be life-threatening. It can also lead to obstruction and incarceration symptoms of the herniated organs, such as vomiting and other gastrointestinal symptoms (7). In right-sided Bochdalek hernia, the contents are mainly the liver, kidney, and fat. Left-sided hernias may include the intestines, spleen, liver, pancreas, kidney or fat (9).

The ITKs are usually normally developed and with good function. In a minority of cases, they may be accompanied by congenital developmental abnormalities, such as anomalies in the origin of renal blood vessels, obstruction at the connection between the renal pelvis and ureter, duplication of the renal pelvis and ureter, and abnormal positioning (10). Most patients with intrathoracic kidneys, especially those with pure ITK, typically show no significant physical signs and are often discovered incidentally. There have been reports that adult patients with intrathoracic kidneys may experience renal insufficiency, urinary system infections, hydronephrosis, kidney stones and other symptoms, but it is not clear whether these are related to the kidney's displacement. Children's ITK with a Bochdalek hernia are often identified in the neonatal or infantile period and may present with respiratory system symptoms such as pneumonia, or digestive system symptoms like vomiting and abdominal pain (5, 11).

ITK can be identified as early as during prenatal check-ups. Some studies have diagnosed ITK by tracking the ectopic renal artery that originates from the aorta and travels upwards through prenatal ultrasound examinations. The absence of the diaphragm and the presence of colon hernia can be detected using MRI, although the diagnostic value of this approach is limited (12, 13). Most patients show lung opacity or a mass on chest x-ray examinations (14). There are reports of patients misdiagnosed with bacterial pneumonia due to cough and fever with left lower lung opacity on chest x-ray, and subsequently treated with antibiotics (15). In the past, when ITK was suspected, scholars confirmed it using magnetic resonance urography or intravenous urography. Nowadays, chest CT typically clearly displays the abnormally positioned kidney and the extent of the diaphragmatic defect, facilitating the diagnosis of ITK and CDH and the formulation of a treatment plan (16–18). When children have a combination of CDH, a gastrointestinal contrast study can be performed to further ascertain the specific conditions of the hernia contents, which may include the stomach, transverse colon, spleen et al. (9).

Intrathoracic kidneys usually do not have obvious symptoms and do not require special treatment (4). When patients present with symptoms in the digestive, respiratory, or urinary systems, a comprehensive assessment must be made, considering the surgical risks and therapeutic benefits. It is currently believed that when ITK is combined with CDH, surgical assessment and intervention should be considered (4). The assessment includes evaluating the severity of the child's symptoms, the risk of the hernia contents becoming incarcerated, and the surgical risks, especially in neonates and infants. The prognosis for children after surgical treatment is generally good. There are multiple methods for the surgical treatment of ITK combined with CDH. Traditional approaches such as open thoracic surgery for CDH are now often enhanced with endoscopic technology for further treatment. Patients typically experience less pain, shorter hospital stays, reduced operation times and lower costs (19). However, the use of thoracoscopy or laparoscopy in the treatment of ITK combined with CDH still needs further exploration. Some scholars use thoracoscopy alone, while others use a combination of thoracoscopy and laparoscopy, both achieving satisfactory therapeutic outcomes with normal kidney function and a better prognosis (20–23). Others simply repair the diaphragmatic hernia and permanently retain the kidney in the thoracic cavity, and the child has a good prognosis after continuous follow-up (24). We present the case of a 6-month-old infant with an ITK combined with a large CDH, who displayed clear symptoms of the gastrointestinal tract and fever. Following comprehensive evaluation, the child underwent successful surgical treatment using laparoscopy, with a favorable prognosis.

## 4 Patient perspective

The parents of the boy were deeply involved throughout the entire treatment and follow-up process. We had detailed conversations with them before, during and after the surgery. They expressed shock at the rarity of their child's disease and were satisfied with the treatment process and surgical outcomes. They believe their child is currently pretty healthy. The mother is eagerly looking forward to more research from doctors around the world to clarify the pathogenesis and genetic aspects of ITK, while also providing guidance on fertility to prevent potential impacts on future generations.

## 5 Conclusions

The co-occurrence of infantile ITK and a large CDH is quite uncommon. Surgical treatment is necessary when symptoms of pneumonia, digestive system issues, or urogenital tract problems are present. Performing the reduction of the ectopic kidney and the repair of the large diaphragmatic hernia through laparoscopy is a minimally invasive and efficacious surgical method.

## Data Availability

The original contributions presented in the study are included in the article/Supplementary Material, further inquiries can be directed to the corresponding author.
